# Trimethylamine N-oxide Supplementation Abolishes the Cardioprotective Effects of Voluntary Exercise in Mice Fed a Western Diet

**DOI:** 10.3389/fphys.2017.00944

**Published:** 2017-11-23

**Authors:** Hongqi Zhang, Jian Meng, Haiyan Yu

**Affiliations:** Department of Anesthesiology, Jining First People's Hospital, Jining, China

**Keywords:** voluntary exercise, western diet, trimethylamine N-oxide, cardiac function, fibrosis

## Abstract

Excessive consumption of western diet (WD) induces obesity, resulting in cardiac dysfunction. Voluntary exercise ameliorates WD-induced obesity, but its effect on cardiac dysfunction remains unclear. Recent evidence suggests that elevated trimethylamine N-oxide (TMAO), a gut microbe-derived metabolite, can impair cardiac function in WD-induced obesity. We hypothesized that cardiac dysfunction in WD-induced obesity would be prevented by voluntary exercise but abolished by TMAO supplementation. Male CD1 mice fed a WD were assigned to sedentary, exercise or exercise with TMAO treatment for 8 weeks. Male CD1 mice fed a normal diet (ND) for 8 weeks were assigned to sedentary (control). Compared with ND-sedentary mice, WD-sedentary mice gained significantly more body weight and displayed metabolic abnormalities at the end of the experiment. Echocardiography showed significantly impaired cardiac systolic and diastolic function in WD-induced obese mice. Voluntary exercise partially attenuated weight gain and metabolic disorders, but completely prevented cardiac dysfunction in WD-induced obese mice. Molecular studies revealed that WD-sedentary mice had elevated plasma TMAO levels, along with increased myocardial inflammation and fibrosis, all of which were inhibited by voluntary exercise. Of note, concomitant administration of TMAO had no effects on body weight and metabolic disorders, but it abolished the beneficial effects of voluntary exercise on cardiac dysfunction, myocardial inflammation, and fibrosis in WD-induced obese mice. The results suggest that voluntary exercise prevents cardiac dysfunction in WD-induced obesity by inhibiting myocardial inflammation and fibrosis. Moreover, the cardioprotective effects of voluntary exercise in WD-induced obesity can be abolished by TMAO supplementation, which abrogates voluntary exercise-induced changes in myocardial inflammation and fibrosis.

## Introduction

Obesity has become a global health problem that is reaching epidemic proportions, regardless of gender and age (Flegal et al., 2012; Head, 2015). Obesity is a major risk factor for the development of many cardiovascular diseases, such as coronary heart disease, heart failure (HF), and stroke (Poirier et al., 2006; Zalesin et al., 2011; Pandey et al., 2015; Aune et al., 2016; Obokata et al., 2017). A clinical study recently revealed that the associations of incident HF with overweight status and obesity are stronger than those for other cardiovascular disease subtypes and is unexplained by traditional risk factors (Ndumele et al., 2016). In addition, several cohort studies showed that almost 60% of heart failure patients are overweight and that 35% are obese (Gustafsson et al., 2005). Although genetic factors are known to play an important role in the development of obesity, excessive consumption of a Western diet (WD), which is high in fat and sugar, and physical inactivity are also considered to contribute to the increasing prevalence of obesity (Sahoo et al., 2015). Experimental studies have demonstrated that WD-induced obese animals exhibit cardiac dysfunction (Carbone et al., 2015; Kesherwani et al., 2015).

Accumulating evidence suggests that trimethylamine N-oxide (TMAO), one of the gut microbiota-derived metabolites, is involved in the pathogenesis of cardiovascular disease (Tang and Hazen, 2014; Brown and Hazen, 2017). We recently reported that consumption of WD elevates TMAO levels in the circulation, which cause myocardial inflammation and fibrosis, resulting in impaired cardiac function in mice (Chen et al., 2017). Thus, interventions to reduce circulating TMAO levels are an important strategy for prevention and treatment of cardiac dysfunction in WD-induced obesity. Unfortunately, currently there are no clinically available drugs that specifically target TMAO.

Exercise has been shown to have many beneficial effects on cardiac function. Exercise can improve cardiac function in the failing heart in both human and animals (Gielen et al., 2015; Natali et al., 2015; Zilinski et al., 2015). Furthermore, exercise ameliorates WD-induced cardiac dysfunction in mice (Kesherwani et al., 2015). However, the molecular mechanism by which exercise improves cardiac dysfunction in WD-induced obesity has yet to be fully elucidated. In animal models, forced exercise is stressful and can cause gut inflammation (Cook et al., 2013) or exacerbate heart failure (Holloway et al., 2015), suggesting that voluntary exercise may be a better model. It has been demonstrated that voluntary exercise ameliorates obesity and metabolic disorders (Bradley et al., 2008; Liu et al., 2015), but data on the effect of voluntary exercise on cardiac dysfunction in WD-induced obesity remain limited. Here, we hypothesized that cardiac dysfunction in WD-induced obesity would be prevented by voluntary exercise and this would be abolished by TMAO supplementation.

## Methods

### Animals

Eight-week-old male CD1 mice were obtained from Vital River (A Charles River Company, Beijing, China). All animals were housed under 12-h light−12-h dark conditions and given free access to food and water. All experimental procedures and protocols used in this study were approved by the Institutional Animal Care and Use Committee of Jining First People's Hospital, and were performed in accordance with the “Guiding Principles for Research Involving Animals and Human Beings” (World Medical Association American Physiological Society, 2002).

### Experimental protocol

To assess the effect of voluntary exercise on WD-induced cardiac dysfunction and molecular mechanism, mice were randomly divided into four groups (*n* = 10 for each group): (1) normal diet-sedentary (ND-SED), (2) western diet-sedentary (WD-SED), (3) western diet-voluntary exercise (WD-EXE), and (4) western diet-voluntary exercise with TMAO administration (WD-EXE-TMAO). Groups underwent simultaneous diet modification and voluntary exercise training with or without administration of TMAO (120 mg/kg) in drinking water (Makrecka-Kuka et al., 2017) for 8 weeks. The ND (Teklad LM-385, Harlan) contained 17% total fat, 0.8% saturated fat, and 0% sucrose, while WD (TD 88137, Harlan) had 42% total fat, 12.8% saturated fat, and 30% sucrose. This WD has been demonstrated to cause cardiac systolic and diastolic dysfunction in mice after 8 weeks of feeding (Carbone et al., 2015; Chen et al., 2017). Mice in the exercise condition were individually housed in cages with free access to telemetered running wheels (Respironics, Bend, OR). Wheel distance was continuously monitored and recorded every hour using a computerized system (VitalView software, Respironics, Bend, OR) and the running distance per 24 h was calculated. Mice in the sedentary condition were individually housed in cages without running wheels. Since mice climb in locked wheels, we deliberately did not place sedentary mice in cages with locked wheels to limit physical activity. Food intake and body weight were measured weekly. At baseline and 8 weeks after WD feeding and exercise, a tail-cuff plethysmography (BP-98A; Softron Co, Tokyo, Japan) was used to measure arterial blood pressure and heart rate, and an echocardiogram was performed to assess cardiac function. At the termination of the experiment, animals were fasted overnight before sacrifice. Plasma samples were collected for biochemical measurements, hearts were rapidly harvested and heart tissue was fixed in 10% formalin to determine myocardial fibrosis, or immediately frozen in liquid nitrogen and stored at −80°C for further molecular analysis.

### Echocardiography

Transthoracic echocardiogram (Sonos 5500, Philips Medical Systems, Andover, MA, USA) was performed to assess cardiac function, as previously described (Chen et al., 2017). Briefly, mice were lightly anesthetized with 2% isoflurane and placed on a heating table in a supine position. Changes in left ventricular (LV) dimensions, mass, ejection fraction (EF), and cardiac output (CO) were determined by M-mode and two-dimensional echocardiography. For determining the isovolumetric contraction time (ICT) and relaxation time (IRT), the ejection time, and the myocardial performance index (MPI), an apical four-chamber view of the left ventricle was obtained and a pulsed-wave Doppler system was performed. Doppler time intervals were determined from mitral inflow and left ventricle outflow Doppler tracings. Three time-periods were measured: ICT from the end of the diastolic early filling (E)/atrial contraction (A) waveform to the beginning of the aortic flow, the ejection time from the beginning to the end of the aortic flow, and the IRT from the end of the aortic flow to the beginning of the E/A waveform. MPI is defined as the sum of ICT and IRT divided by ejection time.

### Assessment of plasma TMAO

Plasma TMAO levels were analyzed by liquid chromatography coupled with triple-quadrupole mass spectrometry as described previously (Chen et al., 2017).

### Western blot analysis

Western blot was used to analyze protein levels of vimentin and pro-inflammatory cytokines tumor necrosis factor (TNF)-α and anti-inflammatory cytokine IL-10 in the heart, as previously described (Chen et al., 2017). Briefly, the heart tissue was homogenized in ice cold lysis buffer with protease inhibitor and a Bradford assay was used to determine the protein concentration. Proteins were fractionated on SDS-PAGE (12% polyacrylamide gels), transfer to polyvinyl difluoride membrane, and were probed for protein expression by immunoblotting. The primary antibodies were vimentin (Santa Cruz Biotechnology, Santa Cruz, CA, USA), TNF-α (Cell Signaling Technology, Beverly, MA, USA), IL-10 (EMD Millipore Corporation, Billerica, MA, USA), and β-actin (Santa Cruz Biotechnology Inc, Santa Cruz, CA, USA). Antibody binding was detected with horseradish peroxidase-conjugated second antibody (Santa Cruz Biotechnology Inc, Santa Cruz, CA, USA). Band intensities were quantified by ImageJ software (National Institutes of Health, Bethesda, Maryland, USA). All data were normalized to b-actin.

### Assessment of myocardial fibrosis

Masson Trichrome staining was performed to assess myocardial fibrosis as previously described (Chen et al., 2017). Briefly, heart tissues were cut into 18-μm sections and mounted on glass slides. The sections were incubated in Bouin's fluid for 1 h followed by staining with Biebrich scarlet-acid fuchsin for 10 min. Before staining by aniline blue, the sections were incubated in phosphotungstic-phosphomolybdic acid solution for 15 min. After rinsing briefly in deionized water, the sections were placed in 1% acetic acid solution for 1 min, rinsed, dehydrated, mounted, and imaged using a light microscope. Myocardial interstitial fibrosis in the sections was calculated based upon percentages of collagen positive areas in the total myocardial area.

### Biochemical assay

A glucose analyzer (Prestige Smart System) was used to measure the levels of plasma glucose. The levels of plasma cholesterol and triglycerides were determined by ELISA using commercially available kits (Pointe Scientific, Canton, MI, USA).

### Statistical analysis

The results are presented as mean ± SE. Data for body weight, echocardiographic and hemodynamic (blood pressure and heart rate) parameters were analyzed using two-way ANOVA followed by a multiple-comparison test. The rest of the data were analyzed using one-way ANOVA followed by a multiple-comparison test. Spearman correlation was used to examine the associations between circulating TMAO and the markers of cardiac function or protein levels of vimentin in the heart. Differences were considered significant if *P* < 0.05.

## Results

### Effects of voluntary exercise and TMAO supplementation on body weight and metabolic disorders

**Figure 1:**
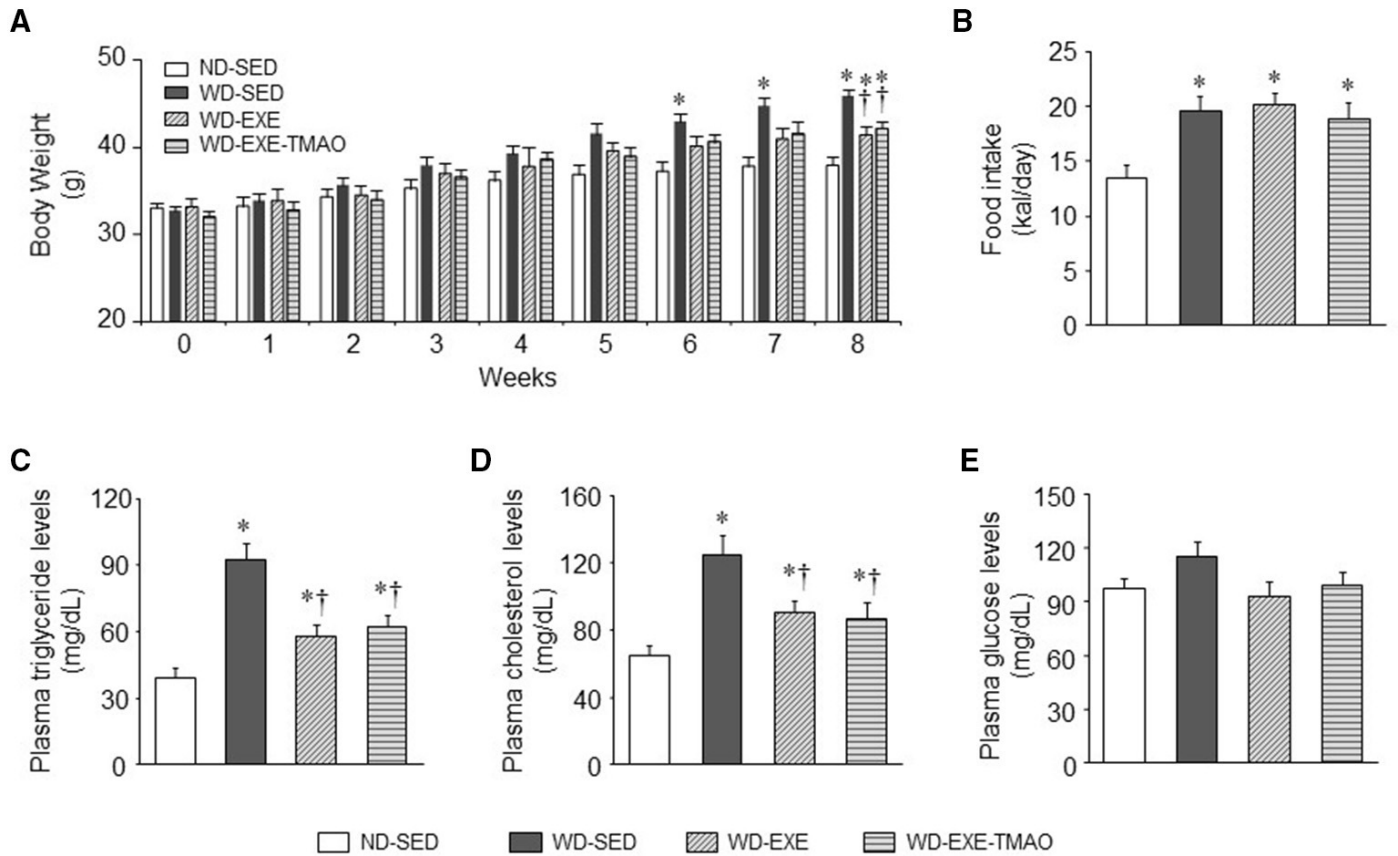
Effects of voluntary exercise and TMAO supplementation on body weight, food intake, and metabolic disorders in mice fed a western diet (WD). Compared with normal diet (ND)-sedentary mice, WD-sedentary mice gained significantly more body weight **(A)** had increased daily caloric intake **(B)** and developed dyslipidemia **(C,D)** after 8 weeks of feeding. Voluntary exercise did not alter daily caloric intake, but partially attenuated weight gain and dyslipidemia. TMAO administration had no effects on these measured parameters in mice fed a WD. There were no differences in plasma glucose levels among the four groups **(E)**. Sedentary: SED; voluntary exercise: EXE. Data are presented as mean ± SE (*n* = 10 for each group). ^*^P < 0.05 vs. ND-SED; ^†^*P* < 0.05 vs. WD-SED.

The WD fed mice treated with daily volitional exercise alone ran an average of 6.14 ± 1.07 km/day, and the WD fed mice treated with daily volitional exercise and TMAO ran an average of 6.20 ± 1.15 km/day. There was no difference in daily running distance between 2 exercise groups. Body weight was similar among the four groups at baseline (before WD feeding and exercise) (Figure 1A). After 6 weeks of WD feeding, WD-sedentary mice showed a significant increase in body weight compared with ND-sedentary mice and this trend continued throughout the dietary protocol. The daily caloric intake was significantly higher in WD-sedentary mice than ND-sedentary mice (Figure 1B). At the end of the experimental protocol (8 weeks after WD feeding and exercise), the levels of plasma triglyceride and cholesterol were significantly higher in WD-sedentary mice when compared with ND-sedentary mice (Figures 1C,D). These data indicate that mice receiving a WD for 8 weeks develop obesity and metabolic disorders. Voluntary exercise in WD-induced obese mice did not alter daily caloric intake, but significantly reduced body weight gain and decreased the levels of plasma triglyceride and cholesterol. Of note, body weight and above metabolic parameters were still significantly higher in these obese mice that received voluntary exercise than ND-sedentary mice. In addition, concomitant administration of TMAO had no effects on body weight, daily caloric intake, and metabolic parameters in WD-induced obese mice. Plasma glucose levels were similar across all four groups at 8 weeks (Figure 1E).

### Effects of voluntary exercise and TMAO supplementation on cardiac dysfunction in WD-induced obesity

**Figure 2:**
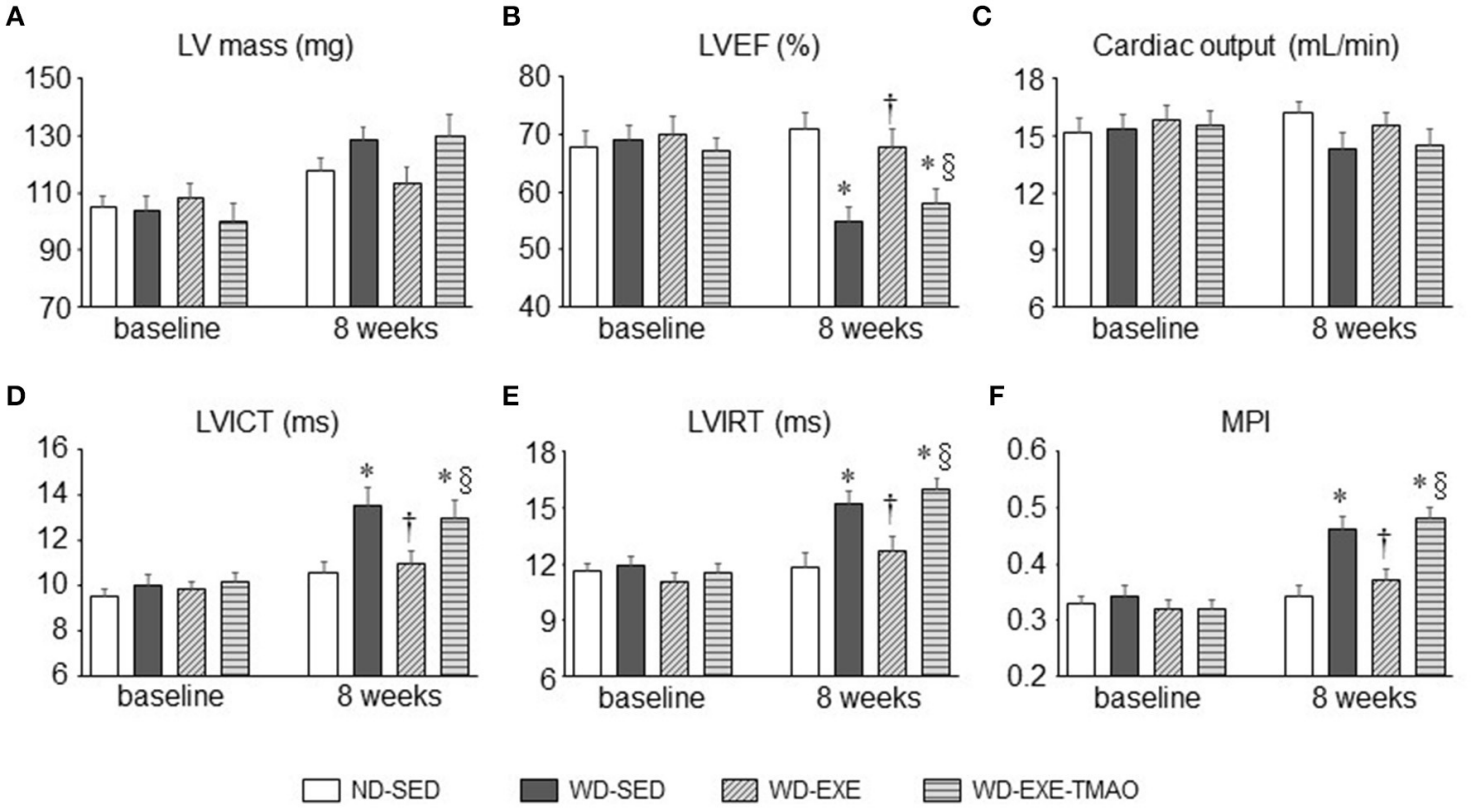
Effects of voluntary exercise and TMAO supplementation on LV mass and cardiac function in mice fed a western diet (WD). Compared with normal diet (ND)-sedentary mice, WD-sedentary mice had impaired systolic **(B)** and diastolic function **(D–F)** at the end of the study protocol (8 weeks after feeding). Voluntary exercise, beginning at the start of WD feeding, prevented cardiac dysfunction, whereas concomitant administration of TMAO completely abolished the beneficial effect of voluntary exercise on cardiac dysfunction in mice fed a WD. No differences in LV mass **(A)** and cardiac output **(C)** were observed among the four groups. Sedentary: SED; voluntary exercise: EXE; LV, left ventricular; EF, ejection fraction; ICT, isovolumetric contraction time; IRT, isovolumetric relaxation time; MPI, myocardial performance index. Data are presented as mean ± SE (*n* = 10 for each group). ^*^P < 0.05 vs. ND-SED; ^†^*P* < 0.05, WD-EXE vs. WD-SED; ^§^*P* < 0.05, WD-EXE-TMAO vs. WD-EXE.

Echocardiography showed that the LV mass, cardiac systolic, and diastolic function were comparable across all four groups at baseline (Figure 2). Eight weeks after WD feeding and exercise, WD-sedentary mice exhibited significantly decrease in LVEF by ~22% (Figure 2B) and increases in LVICT (Figure 2D), LVIRT (Figure 2E), and MPI (Figure 2F) by ~27, 29, and 34%, respectively, compared with ND-sedentary mice. Voluntary exercise, beginning at the start of WD feeding, prevented WD-induced changes in LVEF, LVICT, LVIRT, and MPI, whereas combination of exercise and TMAO administration completely abolished the beneficial effects of voluntary exercise on LVEF, LVICT, LVIRT, and MPI in WD-induced obese mice. No differences in LV mass (Figure 2A) and cardiac output (Figure 2C) were observed among the four groups at 8 weeks.

### Effects of voluntary exercise and TMAO supplementation on plasma TMAO levels in WD-induced obesity

**Figure 3:**
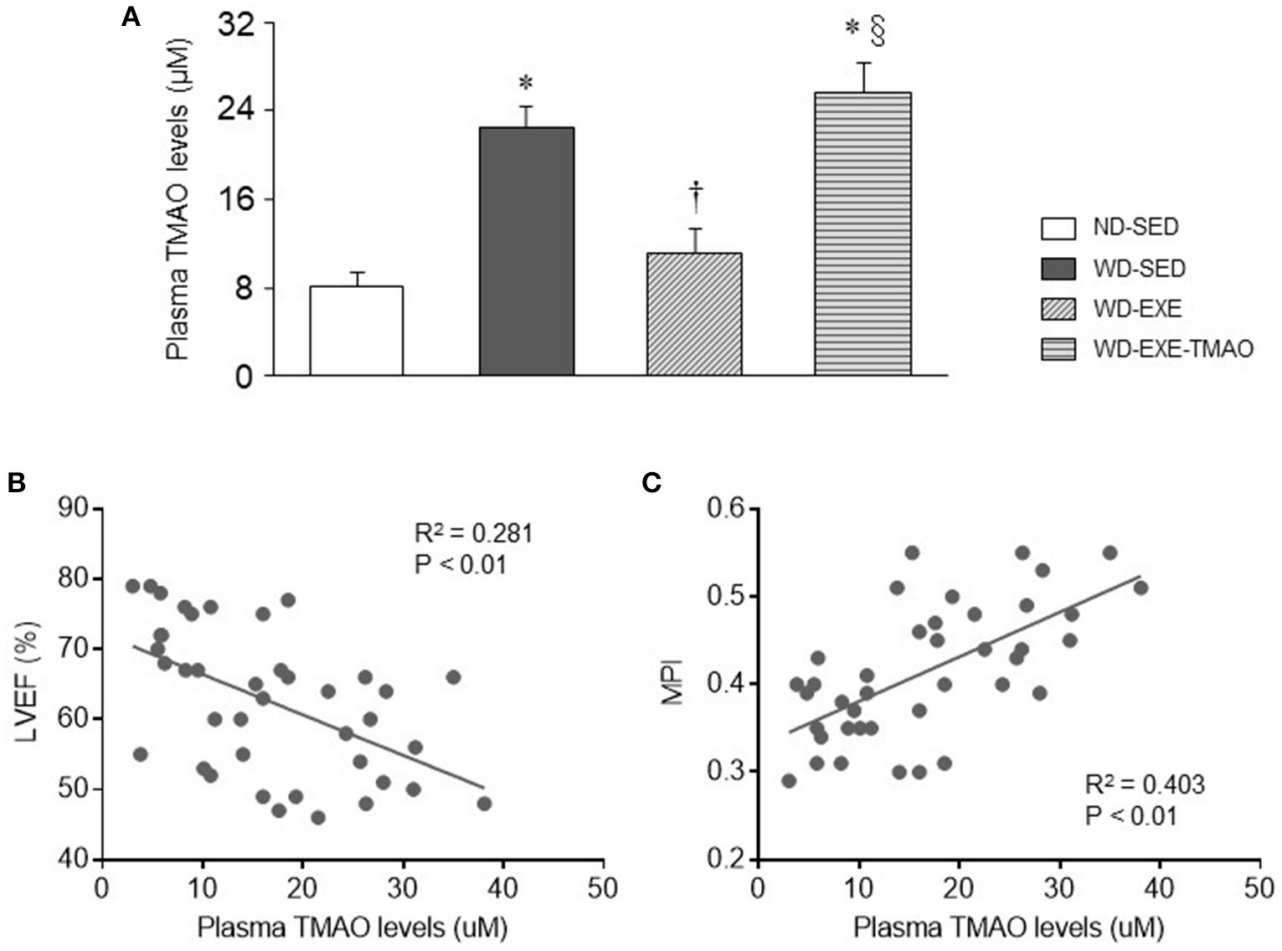
Effects of voluntary exercise and TMAO supplementation on plasma TMAO levels in mice fed a western diet (WD). **(A)** Eight weeks after WD feeding, WD-sedentary mice exhibited elevated plasma TMAO levels as compared to normal diet (ND)-sedentary mice. Elevated plasma TMAO levels were inhibited by voluntary exercise but reversed by concomitant administration of TMAO in mice fed a WD. **(B,C)** Plasma TMAO levels were significantly correlated with cardiac parameter LVEF or MPI. Sedentary: SED; voluntary exercise: EXE. Data are presented as mean ± SE (*n* = 10 for each group). ^*^P < 0.05 vs. ND-SED; ^†^*P* < 0.05, WD-EXE vs. WD-SED; ^§^*P* < 0.05, WD-EXE-TMAO vs WD-EXE.

To determine whether voluntary exercise and TMAO supplementation alter circulating TMAO levels, liquid chromatography coupled with triple-quadrupole mass spectrometry was applied to assess plasma TMAO levels. As shown in Figure 3A, 8 weeks after WD feeding and exercise, WD-sedentary mice had markedly higher levels of plasma TMAO compared with ND-sedentary mice. WD-induced elevation in plasma TMAO levels was completely inhibited by voluntary exercise but this inhibitory effect was abrogated by concomitant administration of TMAO. Importantly, plasma TMAO levels were negatively correlated with cardiac parameter LVEF (Figure 3B) and positively correlated with cardiac parameter MPI (Figure 3C).

### Effects of voluntary exercise and TMAO supplementation on myocardial fibrosis in WD-induced obesity

**Figure 4:**
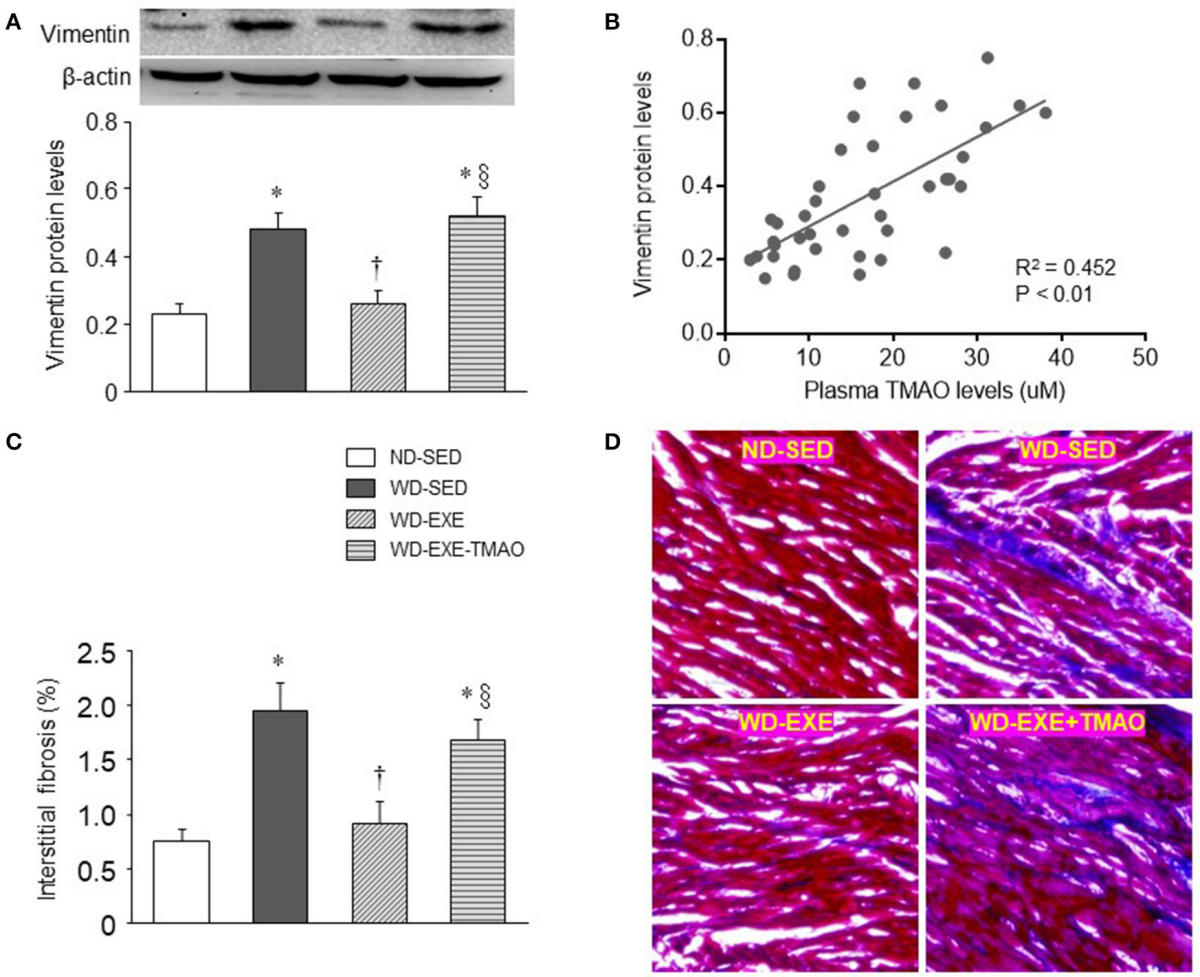
Effects of voluntary exercise and TMAO supplementation on myocardial fibrosis in mice fed a western diet (WD). Compared with normal diet (ND)-sedentary mice, WD-sedentary mice had increased protein levels of vimentin (a marker of fibroblasts, **A)** and interstitial fibrosis **(C)** in the heart, which were prevented by voluntary exercise. Concomitant administration of TMAO abrogated the beneficial effects of voluntary exercise on protein levels of vimentin and interstitial fibrosis. Notably, plasma TMAO levels were significantly correlated with protein levels of vimentin in the heart **(B)**. Representative images of Masson Trichrome staining in heart sections are shown in **(D)**. Sedentary: SED; voluntary exercise: EXE. Data are presented as mean ± SE (*n* = 10 for each group). ^*^P < 0.05 vs. ND-SED; ^†^*P* < 0.05, WD-EXE vs. WD-SED; ^§^*P* < 0.05, WD-EXE-TMAO vs. WD-EXE.

Cardiac fibroblasts are critical in regulation of extracellular matrix synthesis (Fan et al., 2012). Hyperactivity of cardiac fibroblasts can lead to increased production and deposition of extracellular matrix proteins in the myocardium, known as fibrosis (Fan et al., 2012). Elevated TMAO levels have been shown to cause myocardial fibrosis, contributing to impaired cardiac systolic and diastolic function in obese animals induced by HFD (Carbone et al., 2015; Kesherwani et al., 2015; Organ et al., 2016; Chen et al., 2017). To examine whether voluntary exercise prevents activity of cardiac fibroblasts and myocardial fibrosis, we measured protein levels of vimentin, a marker of cardiac fibroblasts, and performed Masson Trichrome staining to assess myocardial interstitial fibrosis at 8 weeks after WD feeding and exercise. Compared with ND-sedentary mice, WD-sedentary mice showed significant increases in protein levels of vimentin (Figure 4A) and interstitial fibrosis (Figures 4C,D) in the heart, which were prevented by voluntary exercise. Of note, the beneficial effects of voluntary exercise on protein levels of vimentin and interstitial fibrosis in the heart were reversed by concomitant administration of TMAO in WD-induced obese mice. Moreover, plasma TMAO levels were positively correlated with protein levels of vimentin in the heart (Figure 4B).

### Effects of voluntary exercise and TMAO supplementation on myocardial inflammatory cytokines in WD-induced obesity

**Figure 5:**
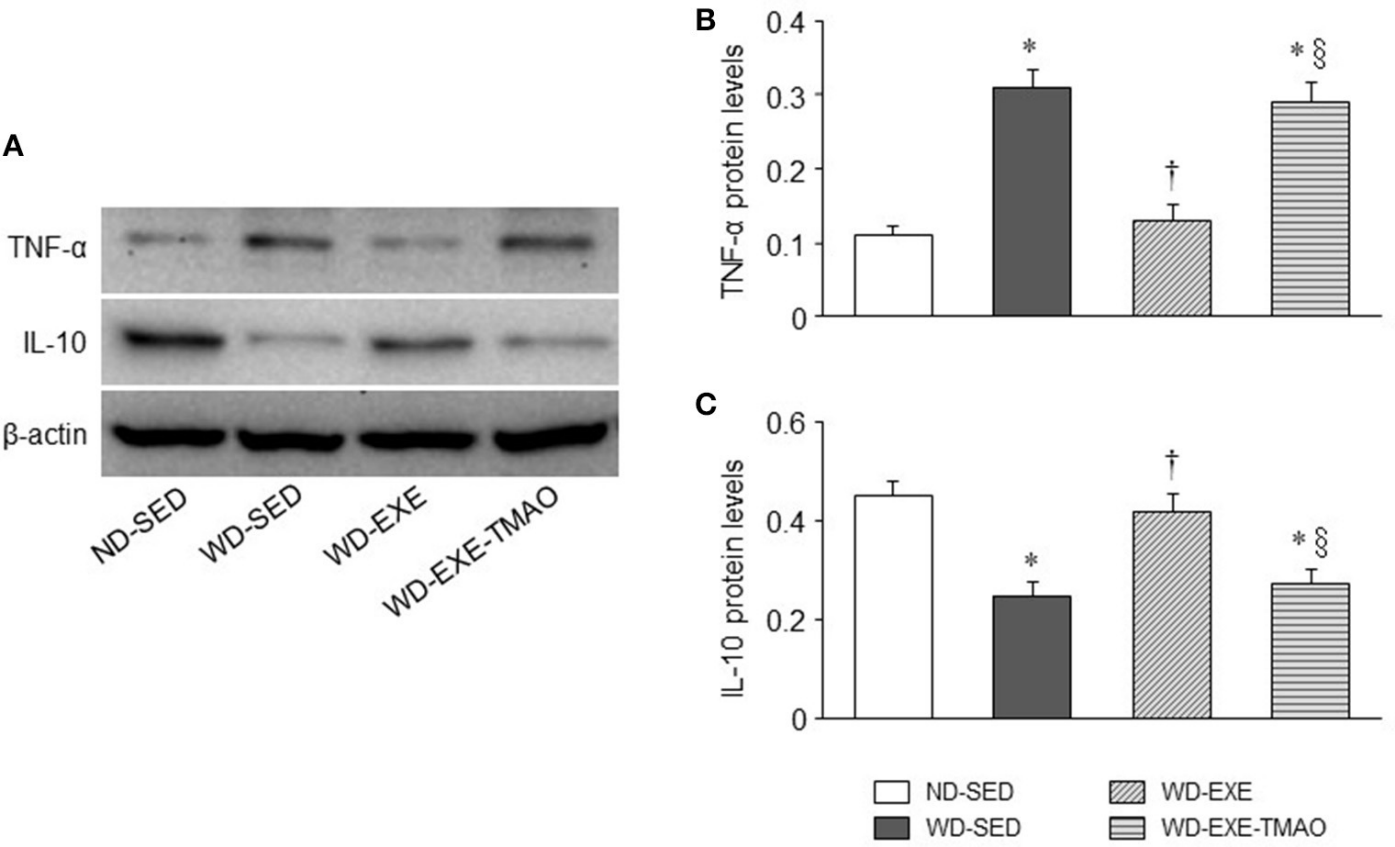
Effects of voluntary exercise and TMAO supplementation on myocardial inflammatory cytokines in mice fed a western diet (WD). **(A)** Representative Western blots of inflammatory cytokines from each group. Compared with normal diet (ND)-sedentary mice, WD-sedentary mice exhibited increased protein levels of pro-inflammatory cytokines tumor necrosis factor (TNF)-α **(B)** and decreased protein levels of anti-inflammatory cytokine IL-10 **(C)**. WD-induced changes in both inflammatory cytokines were prevented by voluntary exercise but reversed by concomitant administration of TMAO in mice fed a WD. Sedentary: SED; voluntary exercise: EXE. Data are presented as mean ± SE (*n* = 10 for each group). ^*^P < 0.05 vs. ND-SED; ^†^*P* < 0.05, WD-EXE vs. WD-SED; ^§^*P* < 0.05, WD-EXE-TMAO vs. WD-EXE.

Our recent study demonstrated that elevated TMAO induces myocardial inflammation, which may lead to myocardial fibrosis and cardiac dysfunction in WD-induced obese mice (Chen et al., 2017). To determine whether inhibition of plasma TMAO elevation by voluntary exercise prevents myocardial inflammation, we next measured expression of inflammatory cytokines in the hearts. As illustrated in Figure 5, WD-sedentary mice had significantly higher protein levels of TNF-α (a key pro-inflammatory cytokine that contributes to myocardial fibrosis) and lower protein levels of IL-10 (an anti-inflammatory cytokine that is involved in the protection against WD-induced inflammation) (Grant et al., 2014) than ND-sedentary mice. WD-induced changes in protein levels of TNF-α and IL-10 were normalized by voluntary exercise. However, concomitant administration of TMAO abolished the effects of voluntary exercise on protein levels of both inflammatory cytokines in WD-induced obese mice.

### Effects of voluntary exercise and TMAO supplementation on blood pressure and heart rate in WD-induced obesity

**Figure 6:**
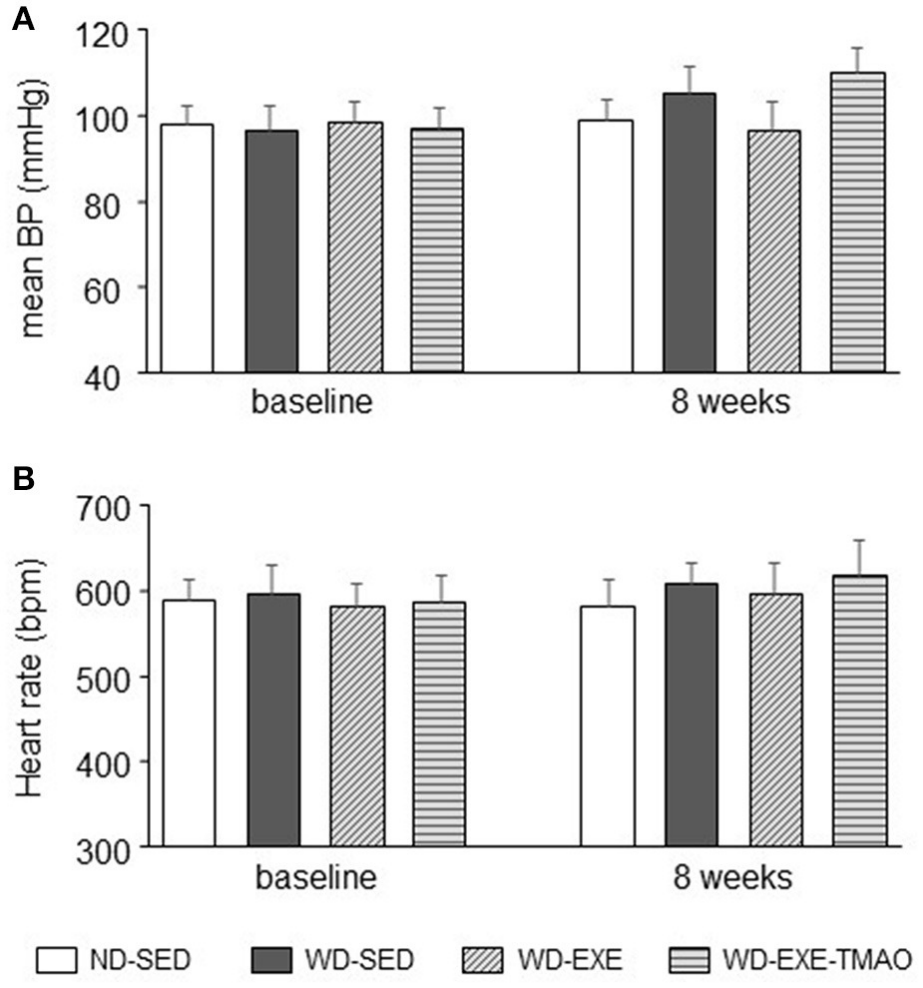
Effects of voluntary exercise and TMAO supplementation on mean blood pressure and heart rate in mice fed a western diet (WD). No significant differences in mean blood pressure (BP), **(A)** and heart rate **(B)** were observed across the four groups at baseline and 8 weeks. Sedentary: SED; voluntary exercise: EXE; TMAO: trimethylamine N-oxide. Data are presented as mean ± SE (*n* = 10 for each group).

The mean blood pressure or heart rate was similar among the four groups at baseline (Figure 6). The mean blood pressure tended to be higher in WD-sedentary mice than ND-sedentary mice at 8 weeks, but the difference between groups did not reach statistical significance (*P* = 0.79). Neither voluntary exercise nor concomitant administration of TMAO altered mean blood pressure in WD-induced obese mice. No difference in heart rate was observed across the four groups at 8 weeks.

## Discussion

The major findings of the present study are as follows: (1) voluntary exercise attenuates but does not normalize WD-induced obesity and metabolic disorders in mice; (2) voluntary exercise in WD-induced obese mice prevents cardiac dysfunction, along with normalization of plasma TMAO levels; (3) voluntary exercise in WD-induced obese mice inhibits myocardial fibrosis and inflammation; (4) administration of TMAO in WD-induced obese mice has no effects on metabolic parameters, but reverses voluntary exercise-induced changes in plasma TMAO levels and abrogates the beneficial effects of voluntary exercise on cardiac function, myocardial fibrosis and inflammation. Taken together, these findings suggest that voluntary exercise prevents cardiac dysfunction in WD-induced obesity, but this cardioprotective effect can be abolished by TMAO supplementation.

Numerous studies have shown that obesity and overweight are important risk factors for the development of cardiac dysfunction and heart failure (Kenchaiah et al., 2002; Baena-Diez et al., 2010; Russo et al., 2011; Kesherwani et al., 2015). Despite major advances in obesity research, current therapeutic strategies specifically directed toward obesity-associated cardiac dysfunction are still lacking. Therefore, the investigation of adjunct therapies that could prevent the development of cardiac dysfunction and heart failure in obese patients is of prime importance from a public health perspective. Exercise has been suggested as an adjunct therapy for many chronic diseases including obesity-associated cardiovascular disease. However, there is emerging evidence that forced exercise or prolonged intense exercise can exacerbate inflammation in a mouse model of colitis (Cook et al., 2013), exhibit opposing effects on markers of heart failure and cardiac remodeling in hypertensive rats (Holloway et al., 2015) and does not attenuate the deleterious cardiovascular consequences associated with overweight and obesity in humans (Lind et al., 2017). These observations suggest that voluntary exercise may be a better strategy for prevention and treatment of obesity-associated cardiovascular disease. To date, the effect of voluntary exercise on WD-induced cardiac dysfunction in animal models remain limited. In the present study, we found that an 8-week WD feeding induced obesity and metabolic disorders in mice, which were associated with impaired cardiac systolic and diastolic function as evidence by decrease in LVEF and increases in LVICT, LVIRT, and MPI. These results are consistent with previous studies from our laboratory (Chen et al., 2017) and others (Carbone et al., 2015; Kesherwani et al., 2015). More importantly, we found that cardiac dysfunction observed in WD-induced obese mice were prevented by voluntary exercise, demonstrating that voluntary exercise is an effective strategy capable of preventing obesity-associated cardiac dysfunction.

Accumulating evidence reveals that TMAO, a gut microbiota dependent metabolite, is associated with the pathogenesis of many cardiovascular diseases (Tang and Hazen, 2014, 2017; Organ et al., 2016; Senthong et al., 2016; Li et al., 2017). Clinical studies have shown that consumption of WD causes elevated circulating TMAO levels in humans (Boutagy et al., 2015a,b). Consistent with clinical findings, we recently demonstrated that WD-induced obese mice have elevated circulating TMAO levels (Chen et al., 2017). Elevated circulating TMAO levels may induce cardiac inflammation by promoting pro-inflammatory cytokines and down-regulating anti-inflammatory cytokines, contributing to myocardial fibrosis and cardiac dysfunction in WD-induced obesity and diabetes mellitus (Sun et al., 2007; Carbone et al., 2015; Kesherwani et al., 2015; Chen et al., 2017). To examine whether the cardioprotective effect of voluntary exercise in WD-induced obesity could be influenced by TMAO supplementation, exogenous TMAO was simultaneously given in drinking water during WD feeding and exercise in mice. Our results showed that WD-induced obese mice exhibited significantly higher plasma TMAO levels at 8 weeks, which were accompanied by increased myocardial fibrosis and inflammation, consistent with our recent report (Chen et al., 2017). Voluntary exercise completely inhibited elevation of plasma TMAO in WD-induced obese mice, along with normalization of myocardial fibrosis and inflammation. Importantly, concomitant administration of TMAO reversed voluntary exercise-induced changes in plasma TMAO levels, resulting in abrogation of voluntary exercise-induced beneficial effects on cardiac function, myocardial fibrosis, and inflammation in WD-induced obese mice. Moreover, plasma TMAO levels were significantly correlated with cardiac parameters LVEF and MPI and protein levels of vimentin. Collectively, these data suggest that voluntary exercise may prevent cardiac dysfunction in WD-induced obesity by inhibiting TMAO-mediated myocardial inflammation and fibrosis, but these beneficial effects can be abrogated by TMAO administration. Previous studies have shown that excessive consumption of WD may influence the capacity of the gut microbiota to produce TMAO from these nutrients (Boutagy et al., 2015b). Additionally, consumption of WD can rapidly change the composition of gut microbiota (David et al., 2014), causing increased production of TMAO (Kitai et al., 2016). We speculate that voluntary exercise may prevent WD-induced alterations in gut microbiota composition and function, thus inhibiting the elevation of circulating TMAO. To our knowledge, this is the first study examining the effect of exercise on circulating TMAO in cardiovascular disease.

Although voluntary exercise ameliorated obesity and metabolic disorders as indicated by attenuating body weight gain and reducing levels of plasma triglyceride and cholesterol in WD-induced obese mice, concomitant administration of TMAO completely abolished voluntary exercise-induced beneficial effect on cardiac function without altering either of these metabolic parameters. In addition, LV mass was comparable at baseline and 8 weeks after WD feeding and exercise between ND-sedentary mice and WD-induced obese mice, which is consistent with previous reports showing that obesity or metabolic disorders did not influence cardiac remodeling (Belke et al., 2000; Carbone et al., 2015). Furthermore, there was no significant difference in blood pressure at baseline and 8 weeks after WD feeding and exercise. Thus, it is unlikely that the beneficial effect of voluntary exercise on cardiac dysfunction is due to improvements in metabolic disorders and blood pressure.

In conclusion, the present study demonstrates that voluntary exercise inhibits elevation of gut microbiota-dependent metabolite TMAO, and reduces myocardial inflammation and fibrosis, thus preventing cardiac dysfunction in WD-induced obesity. However, the cardioprotective effects of voluntary exercise in WD-induced obesity can be abolished by TMAO supplementation, which abrogates voluntary exercise-induced changes in myocardial inflammation and fibrosis.

## Author contributions

Conceived and designed the experiments: HZ and JM; Performed the experiments, Analyzed the data, and Wrote the paper: HZ, JM, and HY.

### Conflict of interest statement

The authors declare that the research was conducted in the absence of any commercial or financial relationships that could be construed as a potential conflict of interest. The reviewer JCG and handling Editor declared their shared affiliation.
